# Analysis of CCR9, CXCR5 and ICOS in Circulating Follicular Helper T Cell-like Populations in Sjögren’s Disease

**DOI:** 10.3390/ijms27041765

**Published:** 2026-02-12

**Authors:** Jose Antonio Garcia-Espinoza, Erika Fabiola López-Villalobos, Mariel García-Chagollán, Jefte Felipe Uribe-Martínez, Santiago Torres-Lizárraga, José Francisco Muñoz-Valle, Gloria Esther Martínez-Bonilla, Sergio Cerpa-Cruz, Claudia Azucena Palafox-Sánchez, Miguel Marín-Rosales, Edith Oregon-Romero

**Affiliations:** 1 Doctorado en Ciencias Biomédicas, Centro Universitario de Ciencias de la Salud, Universidad de Guadalajara, Guadalajara 44340, Jalisco, Mexico; jgarcia58@anahuac.mx; 2 Facultad de Ciencias de la Salud, Universidad Anáhuac, Campus Norte, Huixquilucan 52786, Estado de Mexico, Mexico; 3 Laboratorio de Análisis Clínicos y Bacteriológicos (Vinculación), Centro Universitario de Ciencias Exactas e Ingenierías, Universidad de Guadalajara, Guadalajara 44430, Jalisco, Mexico; 4 Instituto de Investigación en Ciencias Biomédicas, Centro Universitario de Ciencias de la Salud, Universidad de Guadalajara, Guadalajara 44340, Jalisco, Mexico; 5 Servicio de Reumatología, Hospital Civil “Fray Antonio Alcalde”, Guadalajara 44200, Jalisco, Mexico; 6 Servicio de Reumatología, Hospital General de Occidente, Zapopán 45170, Jalisco, Mexico

**Keywords:** Sjögren’s disease, ICOS, CCR9, IL-4, IL-21, cTfh-like cells, autoimmunity

## Abstract

Circulant follicular helper T cells (cTfh) are a specialized subset of CD4^+^ T cells that induce immunoglobulin class switching and antibody secretion in plasma cells through the production of IL-21. To investigate the role of cTfh-like cells in the development of Sjögren’s disease (SjD), we analyzed the circulating Tfh-like cells, their production of IL-21 and IL-4, and the co-expression of ICOS, CXCR5, and CCR9 by flow cytometry, and evaluated their association with clinical characteristics of the disease. Percentages of CD4^+^ IL-21^+^ CXCR5^+^ ICOS^+^ CCR9^+^ IL-4^+^ T cells were analyzed in peripheral blood samples from 20 healthy controls (HCs) and 19 patients with SjD. Serum levels of IL-1β, IL-4, IL-6, IL-21, and sCD40L were assessed using a Luminex assay. Laboratory data included anti-Ro/La antibodies, immunoglobulin levels (IgA and IgG), focus score, disease duration, and ESDDAI/SSDDI scores. Decreased frequencies of CXCR5^+^ IL-21^+^ T cells and CCR9^+^ IL-4^+^ T cells were observed in the peripheral blood of patients with SjD. Heatmap analysis was used to identify correlations between cTfh-like cells and clinical parameters. Elevated proportions of cTfh-like cells were positively correlated with disease severity, inflammatory markers, and autoantibody production. High-dimensional analysis identified distinct subpopulations with differential expression of ICOS, CXCR5, CCR9 and IL-21, suggesting heterogeneity of these cells in SjD and their involvement in disease pathogenesis.

## 1. Introduction

Sjögren’s disease (SjD) is a chronic autoimmune disorder characterized by lymphocytic infiltration of exocrine glands, primarily affecting the salivary and lacrimal glands [1]. This debilitating condition leads to the hallmark symptoms of dry mouth and dry eyes and is frequently accompanied by systemic manifestations or extraglandular manifestations that may involve multiple organs, including the pulmonary, renal, cutaneous, articular, neurological, and hematological systems [1,2,3,4,5,6]. Despite extensive research, the precise mechanisms underlying SjD pathogenesis and disease progression remain incompletely understood.

Recent advances in autoimmunity research have highlighted the role of a specialized subset of CD4^+^ T lymphocytes known as T follicular helper (Tfh) cells [7]. These cells promote germinal center (GC) formation, antibody affinity maturation, and memory B-cell generation and have been implicated in the pathogenesis of several autoimmune diseases [8]. Tfh cells regulate humoral immune responses by providing critical support to B cells within germinal centers and are characterized by the expression of surface markers such as PD-1 and CXCR5, which are essential for their migration and positioning within lymphoid follicles [9]. Weinstein and colleagues (2016) demonstrated that IL-21 promotes the selection of high-affinity B-cell clones, whereas IL-4 production induces robust CD40L expression [10]. In this context, Tfh cells facilitate B-cell recruitment and differentiation within lymph node follicles through the production of IL-21, IL-4, CD40L, and CXCL13 [11,12], thereby enhancing antibody responses. Notably, CD40 has been shown to be constitutively expressed by 30–50% of infiltrating lymphocytes, as well as salivary gland epithelial cells in SjD [13].

Another receptor of interest is CCR9, which plays a key role in directing T-cell migration to gut-associated lymphoid tissue [14,15,16]. Recent studies suggest that CCR9 may also be involved in the trafficking of T cells, including circulating Tfh (cTfh) cells, to the salivary and lacrimal glands in SjD [17]. Helen and colleagues (2011) reported an increased frequency of CCR9^+^ Th cells in the peripheral blood of patients with SjD [18]. More recently, Hinrichs et al. demonstrated higher expression of ICOS in CCR9^+^ Tfh cells, both CXCR5^+^ and CXCR5^−^ subsets [19]. A key molecule associated with Tfh cell function is the inducible T-cell co-stimulator (ICOS), a surface receptor expressed on activated T cells [20,21,22]. ICOS plays a pivotal role in Tfh-B-cell interactions within germinal centers, and engagement of ICOS with its ligand on B cells (ICOSL) delivers essential co-stimulatory signals required for the development and maintenance of effective Tfh cell responses [23].

Emerging evidence indicates that a distinct population of Tfh cells, referred to as circulant T follicular helper (cTfh) cells, may contribute to the pathogenesis of SjD [24,25], as well as other autoimmune diseases such as rheumatoid arthritis (RA) [26], systemic lupus erythematosus (SLE) [27], and juvenile dermatomyositis [28]. For instance, increased frequencies of circulating IL-21-producing CXCR5^+^ ICOS^+^ PD1^+^ T cells have been reported in patients with SjD and shown to correlate with serum IgG levels [29]. A recent study analyzing minor salivary gland cell suspensions by flow cytometry identified Tfh cells as PD-1^+^ ICOS^+^ cells, representing approximately 9% of total CD4^+^ T cells in SjD [30], while similar populations accounted for nearly 25% of synovial CD4^+^ PD-1^+^ ICOS^+^ T cells in RA [26]. Unlike conventional Tfh cells that reside within secondary lymphoid organs, cTfh cells are detected in the peripheral blood of SjD patients [25]. Importantly, cTfh frequencies correlate strongly with disease activity, as assessed by the European League Against Rheumatism Sjogren’s Syndrome Disease Activity Index (ESSDAI), as well as with serum autoantibody titers, including antinuclear antibodies (ANAs) and anti–SSA/Ro 52 antibodies [30].

Understanding the role of cTfh-like cells and the combined involvement of ICOS and CCR9 in SjD may provide valuable insights into the immunological mechanisms driving this autoimmune disease. In the present study, we analyze the co-expression of ICOS, CCR9, IL-21, and IL-4 in circulating follicular T cells from patients with SjD, with a focus on their chemokine receptor profiles and cytokine expression patterns.

## 2. Results

### 2.1. Demographic and Clinical Characteristics

This study included 19 patients with Sjögren’s disease (SjD) and 20 healthy controls (HCs). The mean age of the SjD group was 58 years, with a mean disease duration of 4.84 years. Reduced lacrimal secretion was observed, with a mean value of 0.83 mm/5 min, along with moderate lymphocytic infiltration, averaging 2.23 foci per 4 mm^2^.

Regarding treatment, all patients were receiving pharmacological therapy, including prednisone (15.78%), hydroxychloroquine (42.10%), methotrexate (15.78%), azathioprine (31.57%), and rituximab (10.52%). The demographic and clinical characteristics of the study participants are summarized in Table 1.

### 2.2. ICOS^+^ T Cells Increase in Patients with SjD but Not in CXCR5^+^ IL-21^+^ or IL-4^+^ Subsets

**Figure 1:**
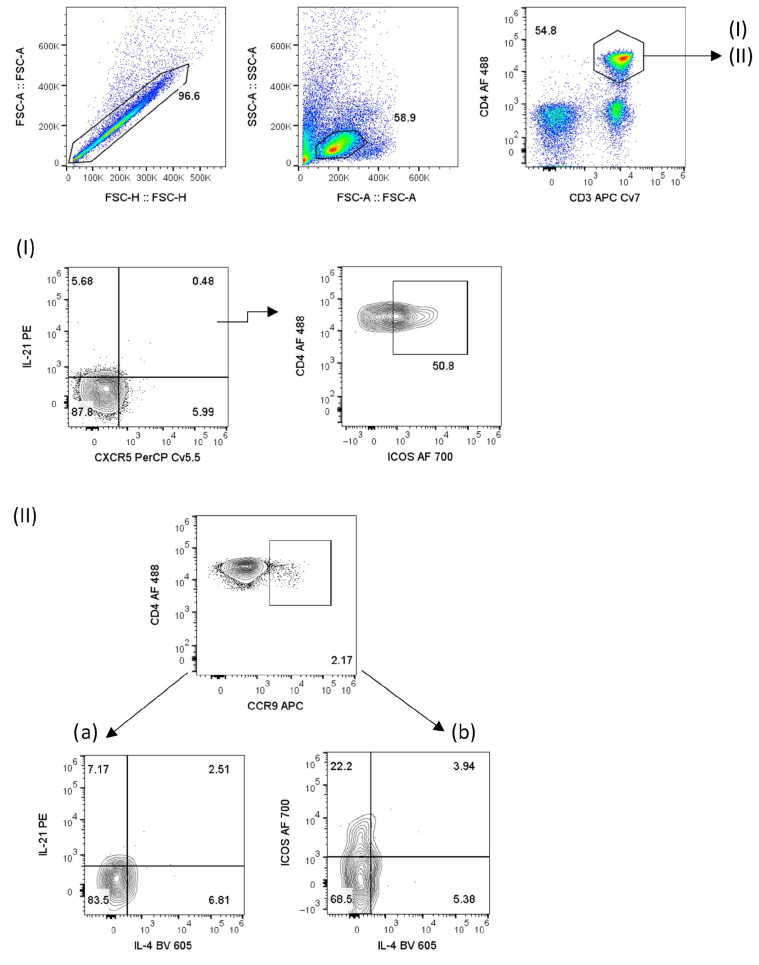
Identification of circulating Tfh-like cells in the peripheral blood of patients with Sjögren’s disease (SjD) and (HC). Representative flow cytometry dot plots showing the gating strategy used to identify T-cell subsets in peripheral blood samples. Two analyses were performed within the CD3^+^ and CD4^+^ T-cell gate: (**I**) the combination of IL-21^+^ CXCR5^+^ ICOS^+^ cells and (**II**) CCR9^+^ cells. Within the CCR9^+^ population, (**a**) IL-21^+^ IL-4^+^ and (**b**) ICOS^+^ IL-4^+^ subsets were evaluated in patients with SjD and healthy controls.

To identify distinct T lymphocyte subpopulations in SjD patients, the analysis strategy shown in Figure 1 was applied using the expression of surface markers CXCR5, ICOS, and CCR9, together with intracellular cytokines IL-4 and IL-21. This approach allowed the identification and quantification of single- and double-positive cell populations.

**Figure 2:**
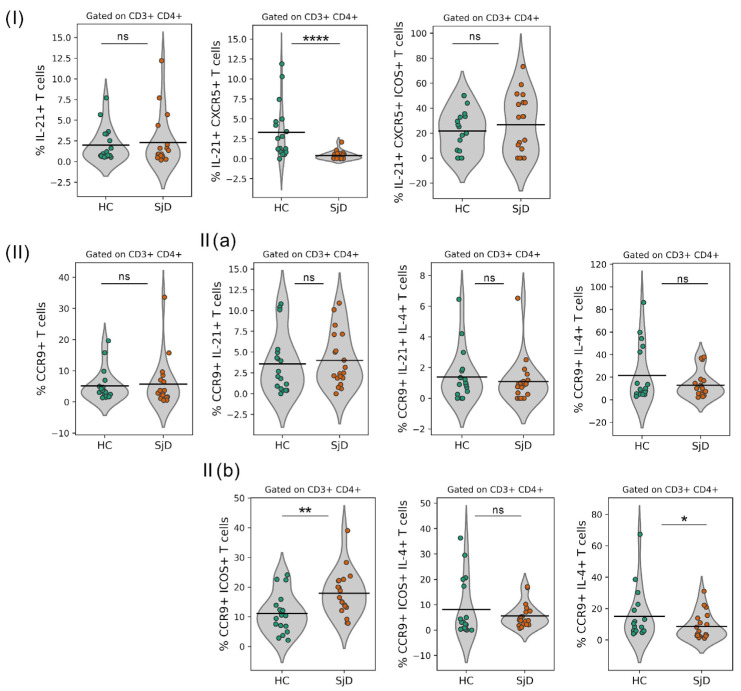
cTfh-like cells are impaired in patients with SjD. (**I**) Representative flow cytometry analysis of peripheral blood from healthy controls (HCs) and patients with Sjögren’s disease (SjD), showing the expression of IL-21, CXCR5, and ICOS among total CD3^+^ CD4^+^ T cells. (**II**) Frequency of CCR9^+^ T cells (left) in the peripheral blood of SjD patients and healthy controls. (**II**(**a**)) Frequency of CD3^+^ CD4^+^ CCR9^+^ IL-21^+^ IL-4^+^ cTfh-like cells in the peripheral blood of HC and SjD patients. (**II**(**b**)) Frequency of CD3^+^ CD4^+^ CCR9^+^ ICOS^+^ IL-4^+^ cTfh-like cells in the peripheral blood of HCs and SjD patients. Values shown in flow cytometry plots represent percentages. Each dot represents an individual subject. Bars indicate the mean (horizontal line) ± SD. Statistical analyses were performed using the nonparametric Mann–Whitney U-test. Data are shown for SjD (n = 19) and HC (n = 20). Statistical significance is indicated as * *p* < 0.05, ** *p* < 0.01, **** *p* < 0.0001; ns, not significant.

The analysis began with CD3^+^ CD4^+^ IL-21^+^ T cells, which were present at similarly low frequencies in healthy controls and patients with SjD (HC: 0.94% vs. SjD: 0.90%, *p* = 0.9612). Within this population, a small subset of CD3^+^ CD4^+^ IL-21^+^ CXCR5^+^ cells—identified as cTfh-like cells—showed a marked and significant reduction in the peripheral blood of SjD patients compared with controls (HC: 2.08% vs. SjD: 0.12%, *p* = 0.0001). In contrast, no significant differences were observed in the triple-positive observed CD3^+^ CD4^+^ IL-21^+^ CXCR5^+^ ICOS^+^ population (HC: 21.10% vs. SjD: 32.70%, *p* = 0.5794) (Figure 2I).

The expression of the chemokine receptor CCR9, previously associated with cTfh-like profiles, was subsequently examined. Notably, the proportion of CD3^+^ CD4^+^ CCR9^+^ ICOS^+^ T cells was significantly higher in SjD patients than in healthy controls (HC: 9.92% vs. SjD: 16.50%, *p* = 0.0033). In contrast, a significant decrease was observed in the CD3^+^ CD4^+^ CCR9^+^ IL-4^+^ subpopulation in SjD patients compared with controls (HC: 10.50% vs. SjD: 3.74%, *p* = 0.0192). This population did not co-express ICOS^+^ but expressed IL-4, a cytokine involved in antibody class switching (Figure 2II(b)).

No significant differences were detected in the frequencies of CD3^+^ CD4^+^ CCR9^+^ cells (HC: 3.69% vs. SjD: 3.0%, *p* = 0.3327), CD3^+^ CD4^+^ CCR9^+^ IL-21^+^ cells (HC: 2.04% vs. SjD: 2.49%, *p* = 0.3122), and CD3^+^ CD4^+^ CCR9^+^ IL-21^+^ IL-4^+^ cells (HC: 0.90% vs. SjD: 0.89%, *p* = 0.5060) (Figure 2II(a)). Similarly, no differences were observed in CD3^+^ CD4^+^ CCR9^+^ ICOS^+^ IL-4^+^ (HC: 2.66% vs. SjD: 3.94%, *p* = 0.4398) (Figure 2II(b)). Overall, the data indicate that although most T cells in SjD patients exhibited increased ICOS expression, this increase was not associated with CXCR5 or IL-4 co-expression.

To identify different T lymphocyte subpopulations in SjD patients, the analysis strategy shown in Figure 1 was applied using the expression of surface markers such as CXCR5, ICOS, and CCR9, together with intracellular cytokines IL-4 and IL-21. This approach allowed the identification of single- and double-positive cell populations. The analysis began with CD3^+^ CD4^+^ IL-21^+^ T cells, which were present at similarly low frequencies in healthy controls and SjD patients (HC: 0.94% vs. SjD: 0.90%, *p* = 0.9612). Within this population, a small subset of CD3^+^ CD4^+^ IL-21^+^ CXCR5^+^ cells—identified as cTfh-like cells—showed a marked and significant decrease in the peripheral blood of SjD patients compared with controls (HC: 2.08% vs. SjD: 0.12%, *p* = 0.0001). In contrast, no significant differences were observed in the triple-positive CD3^+^ CD4^+^ IL-21^+^ CXCR5^+^ ICOS^+^ population (HC: 21.10% vs. SjD: 32.70%, *p* = 0.5794) (Figure 2I).

### 2.3. Serum Levels of cTfh-Associated Cytokines in Patients with SjD

Serum analysis revealed significantly increased levels of inflammatory cytokines in patients with Sjögren’s disease compared with healthy controls. Specifically, IL-1β (HC: 0.24 vs. SjD: 0.80, *p* = 0.0065) and IL-21 (HC: 2.93 vs. SjD: 21.75, *p* = 0.0200) were significantly elevated in the SjD group.

**Figure 3:**
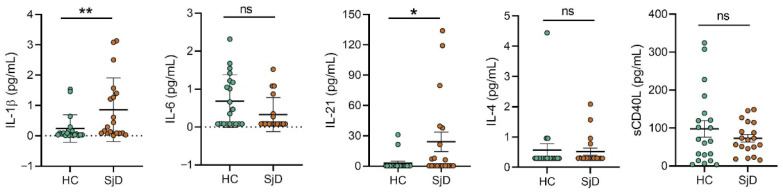
Serum cytokine profile in patients with SjD. Cytokine concentrations in serum samples were quantified using a Bio-Plex Pro^TM^ Human Cytokine 15-Plex Assay. Data are shown for patients with SjD (n = 19) and HCs (n = 20). Bars indicate the mean (horizontal line) ± SD. Statistical analyses were performed using the nonparametric Mann–Whitney U-test. Statistical significance is indicated as * *p* < 0.05, ** *p* < 0.01, ns, not significant.

In contrast, no significant differences were observed between SjD patients and healthy controls for soluble CD40 ligand (sCD40L) (HC: 97.93 vs. SjD: 78.45, *p* = 0.9227), IL-4 (HC: 0.57 vs. SjD: 0.50, *p* = 0.6450), or IL-6 (HC: 0.68 vs. SjD: 0.30, *p* = 0.0670) (Figure 3).

**Figure 4:**
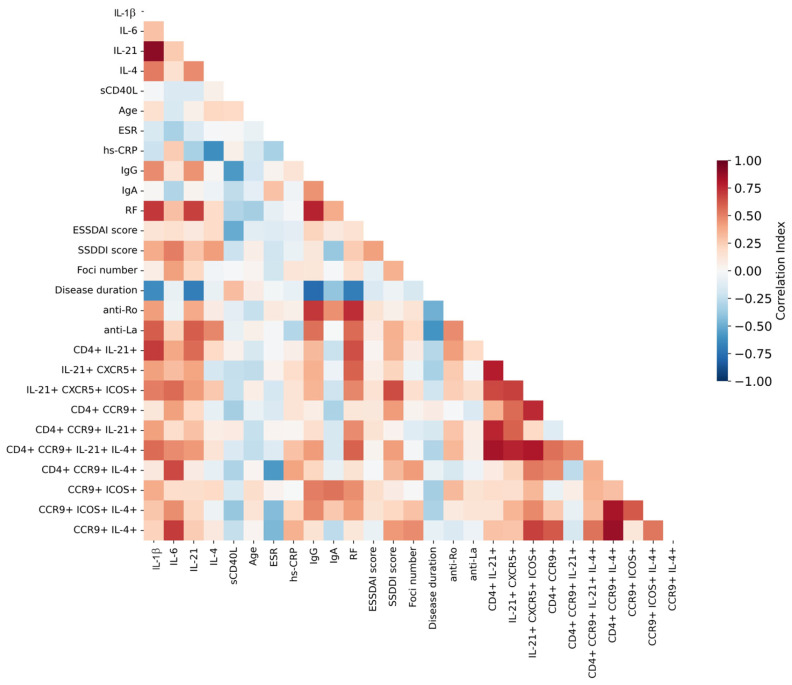
Correlations between T-cell phenotypes and clinical parameters in Sjögren’s disease. Correlation coefficients between T-cell subpopulations and clinical parameters are displayed as a heatmap. Correlations were calculated using Spearman’s rank correlation coefficient (r). The magnitude and direction of the correlations are represented on a color scale ranging from blue (strong positive correlation, r = 1.0) to red (strong negative correlation, r = −1.0), with white indicating no correlation (r = 0). The strength and direction of each correlation are indicated by the corresponding r values.

**Figure A1:**
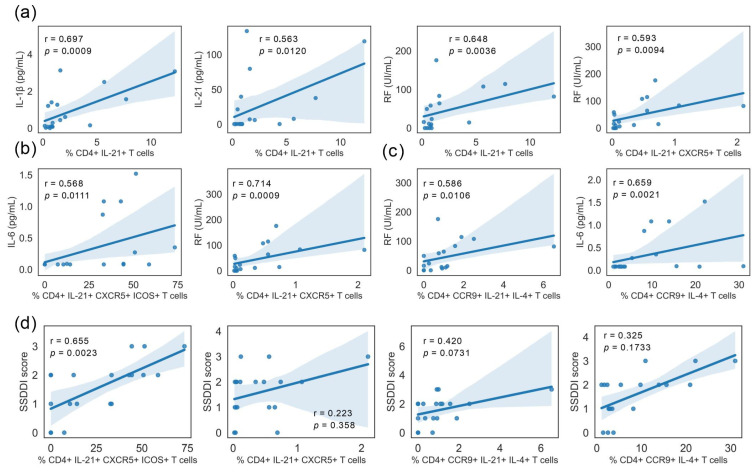
Elevated ratios of cTfh-like cells are positively correlated with disease severity. The most relevant correlations from the heatmap are shown. (**a**) Percentage of CD4^+^ IL-21^+^ cells correlated with IL-1β, IL-21, and rheumatoid factor (RF). (**b**) CD4^+^ IL-21^+^ CXCR5^+^ ICOS^+^ cells correlated with IL-6 and RF. (**c**) Correlation between CD4^+^ CCR9^+^ IL-4^+^ cells and RF and IL-6. (**d**) Correlation between the damage index (SSDDI) and CD4^+^ CXCR5^+^ CCR9^+^ IL-4^+^ subpopulations. Spearman correlation statistics are presented.

An integrated correlation analysis (heatmap) was performed to explore potential associations between inflammatory markers and disease activity. This analysis revealed significant correlations between alterations in follicular T-cell subsets in SjD patients and clinical parameters, including circulating cytokine levels and disease activity indices (Figure 4). Notably, the frequency of CD4^+^ IL-21^+^ T cells showed strong positive correlations with IL-1β levels (IL-1β vs. % CD4^+^ IL-21^+^ T cells: r = 0.69, *p* = 0.0009), IL-21 levels (IL-21 vs. % CD4^+^ IL-21^+^ T cells: r = 0.56, *p* = 0.0120), and rheumatoid factor (RF vs. % CD4^+^ IL-21^+^ T cells: r = 0.64, *p* = 0.0036) (Figure A1a).

Furthermore, the CD4^+^ IL-21^+^ CXCR5^+^ ICOS^+^ cTfh-like population was associated with both IL-6 and RF levels, showing significant correlations with IL-6 (IL-6 vs. % CD4^+^ IL-21^+^ CXCR5^+^ ICOS^+^ T cells; r = 0.56, *p* = 0.0111) and RF (RF vs. % CD4^+^ IL-21^+^ CXCR5^+^ T cells; r = 0.71, *p* = 0.0009) (Figure A1b). Similarly, in the analysis of CCR9-expressing subsets, significant correlations were identified between RF and CD4^+^ CCR9^+^ IL-21^+^ IL-4^+^ ICOS^+^ T cells (RF vs. % CD4^+^ CCR9^+^ IL-21^+^ IL-4^+^ ICOS^+^ T cells; r = 0.58, *p* = 0.0106), as well as between IL-6 and CD4^+^ CCR9^+^ IL-4^+^ T cells (IL-6 vs. % CD4^+^ CCR9^+^ IL-4^+^ T cells; r = 0.65, *p* = 0.0021) (Figure A1c).

Additionally, associations were observed between the SSDDI score and several CD4^+^ T-cell subsets, including CD4^+^ IL-21^+^ CXCR5^+^ ICOS^+^ T cells (SSDDI vs. % CD4^+^ IL-21^+^ CXCR5^+^ ICOS^+^ T cells; r = 0.65, *p* = 0.0023). Weaker or non-significant correlations were observed with CD4^+^ IL-21^+^ CXCR5^+^ T cells (r = 0.23, *p* = 0.358) and CD4^+^ CCR9^+^ IL-21^+^ IL-4^+^ T cells (r = 0.42, *p* = 0.0731). A significant association between IL-6 and CD4^+^ CCR9^+^ IL-4^+^ T cells was also confirmed (r = 0.65, *p* = 0.0021) (Appendix A Figure A1).

Collectively, these data suggest that cTfh-like CD4^+^ T-cell populations expressing IL-21, CXCR5, and ICOS—either ICOS^+^ or ICOS^−^—are associated with clinical markers of inflammation in SjD. Moreover, a distinct CCR9-expressing CD4^+^ T-cell subset appears to be linked to both inflammatory parameters and disease damage, as reflected by SSDDI scores.

### 2.4. High-Dimensional Analysis of CD4 T Cells from SjD and Healthy Controls

**Figure 5:**
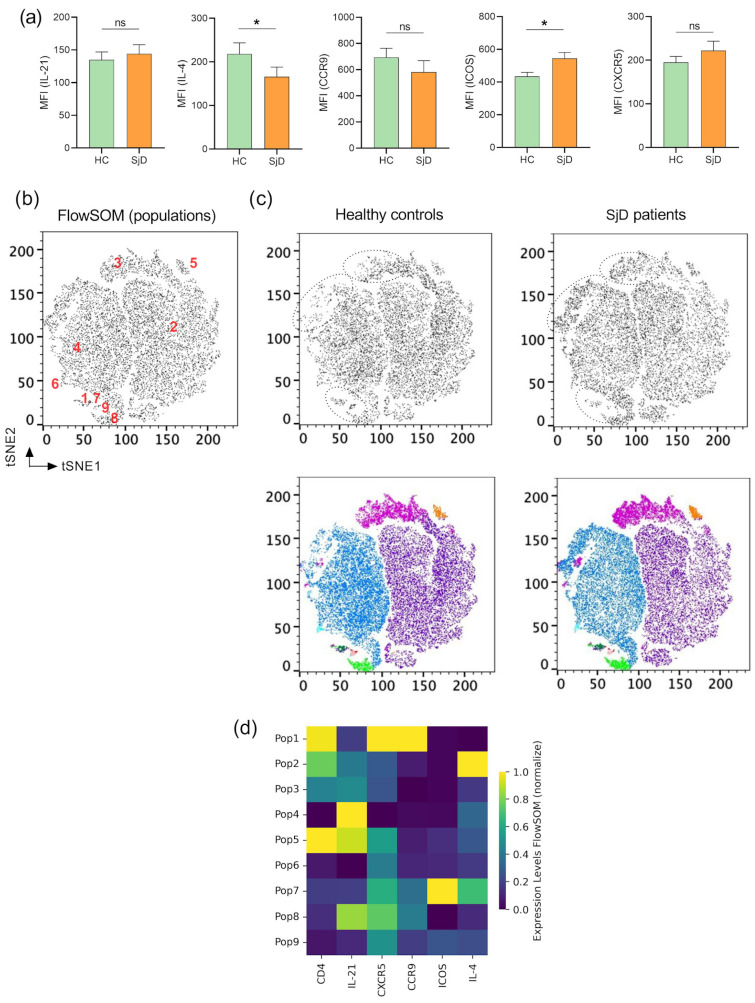
Identification of immunophenotypes among circulating T cells in SjD patients. (**a**) Representation of mean fluorescence intensity (MFI) ± standard error of the mean (SEM) for IL-21, CXCR5, CCR9, ICOS, and IL-4. (**b**) FlowSOM clustering analysis identified nine clusters displayed on a t-SNE plot with different colors (**c**), showing the expression profiles of CD3^+^ T cells using concatenated data from SjD patients and HCs. (**d**) Heatmap representing normalized fluorescence expression levels for each population or cluster identified by FlowSOM. Statistical significance is indicated as * *p* < 0.05; and ns = not significant.

Firstly, the expression of membrane markers (CXCR5, CCR9, and ICOS) and intracellular cytokines (IL-21 and IL-4) in SjD patients and healthy controls (HCs) was assessed using mean fluorescence intensity (MFI). ICOS expression was significantly increased, whereas IL-4 levels were lower in SjD patients. However, no significant differences were observed in IL-21, CXCR5, or CCR9 (Figure 5a).

A more comprehensive analysis was conducted to gain insight into CD4 T-cell populations in SjD patients. The aim was to determine whether the population of cTfh cells expressing CXCR5, IL-21, ICOS, and CCR9 represented a single population or distinct subpopulations. This was addressed through unsupervised high-dimensional analysis using flow cytometry data. t-distributed stochastic neighbor embedding (t-SNE) was performed on CD3^+^ T cells from individual samples, which were then concatenated, followed by FlowSOM clustering to compare the expression of six T-cell markers and delineate the populations identified in each cluster. The tSNE algorithm generated two-dimensional coordinates reflecting phenotypic similarities among single cells derived from peripheral blood mononuclear cells of SjD patients and healthy controls (HCs). These analyses revealed nine populations (clusters) in the t-SNE representation of (Figure 5b).

In this context, no single population was found to express all markers simultaneously, contrary to our initial expectations. In the high-dimensional analysis, cluster 1 showed high expression of CD4, CXCR5, and CCR9, while cluster 2 was predominantly CD4^+^ and primarily expressed IL-4. Although the conventional analysis did not include the CD8 marker, cluster 4 appeared to be CD4^−^ and, notably, exhibited the highest expression of IL-21. Another relevant subset identified through high-dimensional analysis was cluster 7, characterized by co-expression of ICOS and IL-4 (Figure 5d).

## 3. Discussion

One of the most critical events in the humoral immune response is the formation of germinal centers, antibody affinity maturation, and class switching, all of which contribute to autoimmune disease. In the case of SjD, tissue damage, particularly in the salivary glands and eyes, is associated with severe inflammation related to local infiltration of distinct T-cell subsets and acinar atrophy. However, the role of T-cell migration in disease pathogenesis has yet to be fully understood, especially regarding T cells in the salivary gland and peripheral blood.

Since the initial description of circulating CXCR5^+^ CD4^+^ T cells as counterparts of Tfh cells in peripheral blood, their role in regulating humoral immune responses has been well established [28,30,31]. Seminal studies have shown that specialization occurs among different cTfh subsets, influencing their ability to stimulate naïve or memory B cells to differentiate, particularly in SjD.

According to flow cytometry analysis (Figure 1), CD3^+^ CD4^+^ CCR9^+^ ICOS^+^ T cells were increased compared to HCs. However, a decrease in the frequency of cTfh cells defined as CD3^+^ CD4^+^ IL-21^+^ CXCR5^+^ [29], together with CD3^+^ CD4^+^ CCR9^+^ IL-4^+^ T cells, suggests that these populations may represent distinct IL-4-producing T-cell subsets (Figure 2). Helen M. McGuire et al. described an expansion of CD4^+^ CCR9^+^ T cells in the blood of patients with Sjögren’s disease, which are associated with enhanced B-cell interactions and express high levels of IL-21 and transcription factors such as BCL-6 and MAF; however, these cells are distinct from classical Tfh cells [18]. In this context, CCR9 has been proposed to promote retention within the salivary glands to support humoral responses; however, in our study, these cells did not produce IL-21 in peripheral blood (Figure 2II(a)).

A causal relationship between the accumulation of CCR9^+^ Tfh-like cells and salivary gland inflammation has been demonstrated, characterized by elevated ICOS, PD-1, and CD127 (IL-7Rα) expression and IFN-y and CCL5 production [32]. These findings are consistent with in vivo observations showing increased CCL19, CCL25, and CXCL13 expression in salivary gland tissue from SjD patients [33], while other studies have reported average frequencies of PD-1^+^ ICOS^+^ Tfh cells in peripheral blood [30].

Contrary to some reports [34,35], SjD patients in this study showed a decrease in CD3^+^ CD4^+^ IL-21^+^ CXCR5^+^ T cells, suggesting migration of these cells to the salivary gland. Previous reports indicate that PD-1^+^ ICOS^+^ CD45RO^+^ CD4^+^ T cells are increased within the salivary glands containing ectopic lymphoid structures in Sjögren’s disease and produce IL-21, both locally and in peripheral blood [29]. However, this population likely represents tissue-resident Tfh cells.

Furthermore, our data revealed another T-cell subset (CD3^+^ CD4^+^ CCR9^+^ IL-4^+^), suggesting the presence of a distinct helper T-cell population in SjD. Takashi Maehara et al. reported that CD4^+^ CXCR5^+^ IL-4^+^ Tfh cells localize to lymphoid cuffs surrounding germinal centers but are rare in Sjögren’s disease [36], despite their role in immunoglobulin class switching. We hypothesize that CD3^+^ CD4^+^ CCR9^+^ IL-4^+^ T cells may secrete IL-4 within infiltrated tissues and ectopic lymphoid structures, particularly in the salivary glands of SjD patients. However, in situ immunofluorescence studies are required to confirm this hypothesis.

Previous studies have associated elevated cytokine profiles, including IL-1β, IL-4, IL-6, and IL-21, with increased disease severity in SjD, as measured by ESSDAI and SSDDI scores [37]. In the present study, serum levels of IL-1β, IL-4, IL-21, IL-6, and sCD40L were evaluated (Figure 3). No significant differences were observed for IL-4, IL-6, or sCD40L; however, IL-21 and IL-1β levels were significantly increased, consistent with previous reports [38,39]. Xue-yi Li et al. described that CD4^+^ CXCR5^+^ CCR6^+^ CXCR3^−^ T cells produce IL-21 [40]. Additionally, Alqahtani et al. (2023) reported that reduced serum IL-21 levels were associated with fatigue in patients of Arab descent, whereas elevated IL-21 correlated with extraglandular manifestations and ESSDAI ≥ 5 [41], indicating higher disease activity. In our cohort, patients predominantly exhibited glandular and oral involvement suggesting an IL-1β-driven inflammatory process and an IL-21-mediated environment conducive to cTfh activity, promoting T- and B-cell proliferation, differentiation, and memory formation [11,12]. Notably, no evidence of IL-4-mediated class switching or Th2-driven disease progression was observed.

Correlation heatmap analysis demonstrated significant associations between cTfh-like subsets and cytokines, inflammatory markers, and disease activity (Figure 4). CD4^+^ IL-21^+^ T cells correlated with IL-1β, IL-21, and RF (Appendix A Figure A1a), while cTfh-like cells (CD4^+^ IL-21^+^ CXCR5^+^ ICOS^+^) correlated with IL-6 and SSDDI score. Associations with ocular and oral damage were particularly notable, as these manifestations are closely linked to disease severity in SjD (Figure A1b–d). These findings align with those of Fonseca et al., who reported that PD-1^+^ ICOS^+^ Tfh cells correlated with ESSDAI scores and anti-SSA/Ro52 autoantibodies [30]. Collectively, these results suggest that cTfh-like (CD4^+^ IL-21^+^ CXCR5^+^ ICOS^+^) and CD4^+^ CCR9^+^ IL-4^+^ T-cell subsets may contribute directly to SjD pathogenesis, particularly in patients with higher inflammatory activity and elevated ESSDAI and SSDDI scores.

No significant differences were observed in CXCR5, CCR9, or IL-21 expression; however, increased ICOS expression and reduced IL-4 expression were noted (Figure 5a), suggesting enhanced co-stimulatory signaling via ICOS. Transcriptomic [23,42] and microarray studies [29] support these findings, indicating that ICOS may promote effector T-cell and B-cell hyperactivation, hallmarks of SjD associated with hypergammaglobulinemia and autoantibody production. Additionally, Luo et al. describe that ICOS has been linked to metabolic processes within salivary glands, including oxidative phosphorylation, hypoxia, glycolysis, and epithelial–mesenchymal transition, which are critical for glandular function [42]. Thus, targeting cTfh-like cells and ICOS-mediated pathways may have implications for germinal center formation and lymphoma development in SjD.

Consistent with our findings, IL-4 expression was reduced in SjD patients (Figure 5a). Xiangjun Chen et al. reported that IL-4 and related cytokines correlate with reduced tear production and increased ocular damage [43]. Supporting this, Brayer et al. demonstrated that NOD.IL-4^−/−^ mice do not develop xerostomia, highlighting the role of IL-4 in immunoglobulin class switching, particularly the transition from IgM to IgG, via regulation on anti-M3R IgG production [44]. These observations support the hypothesis that salivary gland dysfunction in SjD involves local IL-4-dependent IgG class switching.

High-dimensional multiparametric flow cytometry identified nine peripheral CD4^+^ T-cell clusters (Populations 1–9; Figure 5b,d). Among these, three clusters exhibited distinct expression profiles: population 1 showed high CXCR5 and CCR9 expression with low IL-4 levels; population 7 was characterized by high ICOS expression; and population 4 expressed IL-21 exclusively (Figure 5d). These findings suggest overexpression of ICOS and activation of multiple T-cell populations, including cTfh-like cells. Population 1 may represent a circulating subset with migratory potential toward salivary glands via CCR9, whereas population 7 appears primarily involved in co-stimulation with limited IL-4-mediated class-switching capacity [12].

The possible disturbance of these cells in SjD might also be explained by treatment; Demarchi et al. found that hydroxychloroquine therapy could be associated with lower disease activity (extraglandular manifestations), including arthritis, fatigue, and hypergammaglobulinemia [5]. The study by Gwenny M. Verstappen et al. demonstrated that abatacept reduced the percentages of cTfh cells and that ICOS expression decreased with treatment [37]. However, most patients with SjD were receiving immunomodulatory treatment, mainly hydroxychloroquine (42.10%) and azathioprine (31.57%). Azathioprine may reduce T-cell proliferation, potentially affecting both regulatory T cells and cTfh-like cells, while hydroxychloroquine decreases antigen presentation by APCs. These effects may have contributed to the low disease activity observed in our cohort.

Finally, further investigation of cTfh-like cells and related subsets is warranted to identify key pathogenic mechanisms in SjD. Future studies employing transcriptomics or mass cytometry will be essential to refine the characterization of these populations and their contribution to SjD pathogenesis.

## 4. Materials and Methods

### 4.1. Study Group

A total of 19 SjD patients and 20 healthy controls (HCs) were recruited from the Rheumatology Service of the *Hospital General de Occidente* (Zapopan, Mexico) and *Hospital Civil Fray Antonio Alcalde* (Guadalajara, Mexico). The diagnosis of SjD was established according to 2016 American College of Rheumatology/European League Against Rheumatism classification criteria, and no participants had other autoimmune diseases. Written informed consent was obtained from all subjects (088/19), and the study was approved by the Ethics Committee of *Hospital Civil “Fray Antonio Alcalde”*, Guadalajara, Mexico. All procedures complied with the Declaration of Helsinki. Demographic and clinical data of enrolled subjects are summarized in Table 1. Exclusion criteria included insufficient blood sample volume, hemolyzed samples, or low cell count or viability. Non-inclusion criteria comprised age under 18 years, absence of a confirmed diagnosis of Sjögren’s disease, or pregnancy.

The Sjögren’s Syndrome Disease Activity Index (SSDAI), Sjögren’s Syndrome Disease Damage Index (SSDDI), and EULAR Sjögren’s Syndrome Disease Activity Index (ESSDAI) were evaluated in SjD patients. Anti-Ro and anti-La antibodies (Orgentec Diagnostika GmbH, Mainz, Germany), complete blood chemistry (Cell-Dyn 1700, Abbott Laboratories, Abbott Park, IL, USA), erythrocyte sedimentation rate (ESR; Wintrobe method), C-reactive protein, and rheumatoid factor (turbidimetry; BS120, Mindray, Shenzhen, China) were measured. IgG and IgA levels were determined by turbidimetry (A15 BioSystem; Guadalajara, Mexico). The normal reference range for IgG individuals older than 19 years (700–1600 mg/dL) was adopted for this study [45]. Focus score data from minor salivary gland biopsies were obtained from each patient’s clinical record.

### 4.2. Multiplex Assay

Serum samples from healthy controls and SjD patients were thawed on ice and centrifuged at 3000 rpm for 5 min to remove debris prior to analysis. A custom human cytokine 16-plex Bio-plex^®^ panel (Bio-Rad Laboratories, Hercules, CA, USA; cat. no. 171AA001M) was used, following the manufacturer’s instructions. The cytokines analyzed in this study included IL-1β, IL-6, IL-21, IL-4, and soluble CD40L (sCD40L) to evaluate the cTfh-associated profile.

### 4.3. Flow Cytometry

Peripheral blood mononuclear cells (PBMCs) were isolated by density gradient centrifugation using Ficoll-Paque Plus (GE Healthcare Biosciences, Darmstadt, Germany; cat. no. 17-1440-02) and cryopreserved in 10% DMSO and 40% FBS. PBMCs were adjusted to a final concentration of 1 × 10^6^ cells/mL and incubated for 4 h at 37 °C with 1 µg/mL Brefeldin A (Biolegend, San Diego, CA, USA; cat. no. 420601).

Surface staining was performed for 30 min at room temperature, followed by fixation and intracellular permeabilization staining for 30 min using BD Fix&Perm (cat. no. 562574). For T-cell characterization, 1 × 10^6^ PBMCs were stained with anti-CD3 APC/Cy7 (BioLegend, San Diego, CA, USA; cat. no. 300426), anti-CD4 AF488 (BioLegend; cat. no. 300518), and anti-ICOS AF700 (BioLegend; cat. no. 313528). Additional antibodies included anti-IL-21 PE (BioLegend, San Diego, CA, USA; cat. no. 513004), anti-CXCR5 PerCP/Cy5.5 (BD Biosciences; cat. no. 356910), and anti-IL-4 BV605 (BioLegend, San Diego, CA, USA; cat. no. 502532). Cells were kept on ice prior to acquisition.

Data were acquired using an Attune NxT flow cytometer (Thermo Fisher Scientific, Waltham, MA, USA) and analyzed with FlowJo software version 10.7 (BD). The flowAI plugin [46] was used for automated quality control of FCS files, retaining datasets with >90% good events. Fluorescence minus one (FMO) controls were used to define positive and negative populations.

### 4.4. ELISA

Serum samples were obtained from peripheral blood of SjD patients and HCs using BD Vacutainer^®^ SST^TM^ tubes and stored at −20 °C until use. Anti-SSA (52–60 kDa) and anti-SSB antibody levels were quantified using commercial ELISA kits (Orgentec Diagnostika GmbH, Mainz, Germany; code 508 and 509), following the manufacturer’s instructions. The detection range was 0–200 U/mL, with a sensitivity of 1 U/mL. Cut-off values for anti-Ro and anti-La positivity were defined as <25 U/mL.

### 4.5. High-Dimensional Analysis of Flow Cytometry Data

t-SNE (an unsupervised nonlinear dimensionality reduction algorithm useful for visualizing clusters that represent cells with a similar phenotype) and FlowSOM analyses (a state-of-the-art clustering and visualization technique to classify cells into distinct cell types) were performed using FlowJo software v10.8. CD4^+^ T cells from HCs and SjD patients were concatenated. t-SNE was performed using equal sampling of 5000 cells per FCS file, 1000 iterations, a perplexity of 30, a learning rate of 10 150, and the k-nearest neighbor (KNN) ANNOY algorithm. The markers used to generate t-SNE maps were CD4, IL-21, CXCR5, CCR9, ICOS, and IL-4.

t-SNE results were subsequently analyzed using the flowSOM clustering algorithm [47]. For both HC and SjD groups, self-organizing maps (SOMs) were generated using hierarchical clustering, identifying nine clusters per SOM. FlowJo’s Cluster Explorer tool was used to visualize and interpret clustering patterns within the high-dimensional dataset.

### 4.6. Statistical Analysis

Normality tests were performed to evaluate the distribution of demographic and clinical variables. Parametric and non-parametric data were expressed as mean or median ± standard deviation (SD), as appropriate. Statistical tests were selected based on data distribution.

Spearman’s correlation analysis was used to assess associations between T-cell frequencies and disease-related variables, including the ESSDAI and SSDDI scores. Correlation results were visualized using a heatmap generated using Python (version 6.1.4), employing Scikit-Learn, NumPy, Matplotlib, Pandas, and Seaborn libraries. A *p*-value < 0.05 was considered statistically significant. Additional analyses were performed using GraphPad Prism version 8.0 (GraphPad Software, San Diego, CA, USA).

## 5. Conclusions

The results demonstrate an altered distribution of CD4^+^ CCR9^+^ ICOS^+^, CD4^+^ CCR9^+^ IL-4^+^, and CD4^+^ IL-21^+^ CXCR5^+^ T-cell subsets in SjD compared with healthy controls. High-dimensional analysis revealed differential expression of ICOS, CXCR5, CCR9 and IL-21, suggesting heterogeneity among these populations and supporting their involvement in SjD pathogenesis.

## Figures and Tables

**Table 1:** Demographic and clinical characteristics in Sjögren’s disease (SjD) patients and healthy controls (HCs) ^1^.

	SjD (n = 19)	HC (n = 20)	*p*-Value
Age, years (min–max)	58 (37–72)	50 (21–67)	
Sex (F/M)	19/0	20/0	
Disease duration, years	4.84 ± 4.89 (1–19)	-	
hs-CRP, mg/L	9.56 ± 4.72	4.24 ± 6.50	0.0058
ESR, mm/h	37.2 ± 12.1 (12–58)	24.6 ± 9.70 (8–45)	0.0022
Schirmer test ≤ 5 mm/5 min	0.83 ± 0.40 (0–1)	-	
Foci number ≥ 1 focus/4 mm^2^	2.23 ± 0.94 (0–4.0)	-	
SSDDI score, mean	1.53 ± 0.96 (0–3)	-	
ESSDAI score, mean	4.84 ± 4.10 (0–14)	-	
RF, UI/mL	45.3 ± 50.5	1.59 ± 3.17	0.0002
IgG, mg/dL	1820 ± 868	1343 ± 372	0.0250
IgA, mg/dL	380 ± 99.8	269 ± 99.1	0.0017
Anti-Ro, UI/mL	172 ± 244	-	
Anti-La, UI/mL	58.3 ± 127	-	
Prednisone, n (%)	3 (15.78)	-	
Hydroxychloroquine, n (%)	8 (42.10)	-	
Azathioprine, n (%)	6 (31.57)	-	
Methotrexate, n (%)	3 (15.78)	-	
Rituximab, n (%)	2 (10.52)	-	

^1^ Data are presented as mean ± SD, mean (minimum–maximum) or number of patients and percentage, as appropriate. Abbreviations: hs-CRP, high-sensitivity C-reactive protein; ESR, erythrocyte sedimentation rate; RF, rheumatoid factor; SSDDI, Sjögren’s Disease Damage Index; ESSDAI, EULAR Sjögren’s Syndrome Disease Activity Index. Treatments include monotherapy and combination therapy with immunosuppressive and/or immunomodulatory drugs. -, not evaluated.

## Data Availability

The original contributions presented in this study are included in the article. Further inquiries can be directed to the corresponding authors.

## Abbreviations

The following abbreviations are used in this manuscript:

ANAs	Antinuclear antibodies
cTfh	Circulant follicular helper T cells
CRP	C-reactive protein
CSs	Control subjects
dL	Deciliter
ESR	Erythrocyte sedimentation rate
ESSDAI	EULAR Sjögren’s Syndrome Disease Activity Index
GC	Germinal center
h	Hour
hs-CRP	high-sensitivity C-reactive protein
ICOS	inducible T-cell co-stimulator
ICOSL	inducible T-cell co-stimulator ligand
mg	Milligram
MIF	Median intensity fluorescence
mL	Milliliter
mm	Millimeter
PBMCs	Peripheral blood mononuclear cells
RF	Rheumatoid factor
RT	Room temperature
SD	standard deviation
SjD	Sjögren’s disease
SSDDI	Sjögren’s Syndrome Disease Damage Index
Tfh	T follicular helper cell
U	Unit
